# Harnessing the wealth of Chinese scientific literature: schistosomiasis research and control in China

**DOI:** 10.1186/1742-7622-5-19

**Published:** 2008-09-30

**Authors:** Qin Liu, Li-Guang Tian, Shu-Hua Xiao, Zhen Qi, Peter Steinmann, Tippi K Mak, Jürg Utzinger, Xiao-Nong Zhou

**Affiliations:** 1 National Institute of Parasitic Diseases, Chinese Center for Disease Control and Prevention, Shanghai 200025, PR China; 2 Department of Public Health and Epidemiology, Swiss Tropical Institute, P.O. Box, CH-4002 Basel, Switzerland

## Abstract

The economy of China continues to boom and so have its biomedical research and related publishing activities. Several so-called neglected tropical diseases that are most common in the developing world are still rampant or even emerging in some parts of China. The purpose of this article is to document the significant research potential from the Chinese biomedical bibliographic databases. The research contributions from China in the epidemiology and control of schistosomiasis provide an excellent illustration. We searched two widely used databases, namely China National Knowledge Infrastructure (CNKI) and VIP Information (VIP). Employing the keyword "*Schistosoma*" () and covering the period 1990–2006, we obtained 10,244 hits in the CNKI database and 5,975 in VIP. We examined 10 Chinese biomedical journals that published the highest number of original research articles on schistosomiasis for issues including languages and open access. Although most of the journals are published in Chinese, English abstracts are usually available. Open access to full articles was available in *China Tropical Medicine *in 2005/2006 and is granted by the *Chinese Journal of Parasitology and Parasitic Diseases *since 2003; none of the other journals examined offered open access. We reviewed (i) the discovery and development of antischistosomal drugs, (ii) the progress made with molluscicides and (iii) environmental management for schistosomiasis control in China over the past 20 years. In conclusion, significant research is published in the Chinese literature, which is relevant for local control measures and global scientific knowledge. Open access should be encouraged and language barriers removed so the wealth of Chinese research can be more fully appreciated by the scientific community.

## Introduction

In this thematic series of *Emerging Themes in Epidemiology*, Fung provides a general overview of Chinese biomedical journals, and a focus on specialized journals in epidemiology, preventive medicine and public health [1]. Moreover, Fung examines two of the six core mainland Chinese biomedical bibliographic databases – WanFang Data and iLib – and emphasises that the Chinese interface of Google Scholar provides a convenient entry point to search for Chinese articles. His analysis complements a recent investigation by Xia *et al*. who studied the accessibility, content and form of five major Chinese biomedical databases, namely (i) Biomedical Literature Database (CBM), (ii) Chinese Medical Current Content (CMCC), (iii) China National Knowledge Infrastructure (CNKI) Medicine and Hygiene Section, (iv) VIP Information (VIP), and (v) the above-mentioned WanFang Data [2]. Finally, Fung discusses the prospects and challenges of English language journals published in China, including issues of language publication bias and quality, and concludes that "Chinese journals are a mine of epidemiological information that is yet to be explored by the outside world" [1].

The purpose of our article is to illustrate that the Chinese literature is indeed a treasure house of biomedical knowledge. One of the most prominent examples is the discovery of the antimalarial properties of artemisinin (the active ingredient derived from the herb *Artemisia annua*) in the early 1970s by Chinese scientists [3,4]. Comprehensive investigations of the chemistry and synthesis of artemisinin and its derivatives, assessment of the antimalarial efficacy in different animal models, toxicity and mechanism of action studies, and clinical trials involving over 2000 patients infected with *Plasmodium *were carried out in the 1970s and published in specialized Chinese journals. In 1982, the key findings were summarized and published in English in a special issue of the *Journal of Traditional Chinese Medicine *[5-8]. However, the real significance of these findings only became clear to the clinical and scientific community outside of China in the mid-1980s when a review article entitled "Qinghaosu (artemisinin): an antimalarial drug from China" was published in *Science *[9]. In the meantime, Klayman's review has been cited 878 times (Thomson ISI Web of Science, accessed 20 March 2008), and hundreds of millions of people have been treated with an artemisinin derivative worldwide [10].

Here, we focus on research contributions on the epidemiology and control of schistosomiasis from the Chinese literature. First, we provide a brief overview of the global situation of schistosomiasis, and then summarize the public health significance and achievements made in the control of the "God of Plague" in China over the past half century [11]. We then put forward the most important Chinese journals that publish original research pertaining to schistosomiasis. We also address the issue of accessibility, which is paramount for discovering the resources within the Chinese biomedical literature. For illustrative purposes, two examples are given: (i) the discovery and development of antischistosomal drugs, and (ii) advances made with molluscicides and environmental management for schistosomiasis control. Given China's track record in controlling schistosomiasis japonica [11,12], and in view of new initiatives underway to control schistosomiasis in Africa [13], researchers and policy-makers alike should look towards accessing the long-standing vast Chinese experience to help guide strategies from local to national and global control.

### Chinese databases: the lay of the land

At present, more than 10 Chinese biomedical databases are available online. The two largest and most widely used databases for searching and retrieving scientific information in biomedicine are CNKI and VIP as shown in Table 1. CNKI embodies more than 8,200 journals, of which 2,460 are considered 'core' journals with the highest reputation. Disciplines include the natural sciences (e.g. agriculture and biology), medical sciences, engineering, humanities, philosophy and the social sciences. Most researchers in these fields consider CNKI as the "Chinese PubMed", as it is the most comprehensive database in these disciplines. CNKI is updated continuously and, since April 2006, provides access to all issues for about half of the journals referenced. At present, CNKI includes over 17.5 million citations.

**Table 1:** Overview of the main Chinese biomedical bibliographic databases.

Database	Year launched	Number of journals covered	Open access	Web-site
CNKI	1999	Over 8,200 full-text journals, including 1,707 medical journals	No	
VIP	2000	Over 8,000 full-text journals, including 1,675 medical journals	No	
Wan Fang	2000	Over 8,000 full-text journals, including 1,004 medical journals	No	
CBM	1995	Over 1,600 biomedical journals	No	
CMCC	1994	Over 1,200 biomedical journals	No	

VIP cites articles from more than 8,000 major Chinese and English periodicals that are published in China. Articles are available from the late 1980s onwards and cover natural sciences, including agriculture, medical sciences and engineering, as well as the humanities, philosophy and the social sciences. Whilst CNKI provides access to full text articles in portable document format (PDF) and China Academic Journals (CAJ) format, VIP has full text articles in PDF only.

Access to articles referenced in either of these databases requires a subscription but abstracts may be downloaded without charge. For individual users, CNKI and VIP even accept payment by Chinese mobile phone services and some "pay-per-download" cards. The latter operates in a similar fashion to a telephone card, hence provides an account and password to log in to the database for subsequent downloading of a limited number of full text articles. Institutional subscriptions held by companies, research institutions and universities permit unlimited access to these databases.

To retrieve an article, the databases can be queried by field tags, including subject, title, keyword, abstract, author, affiliation of the corresponding author, journal name, year of publication, and so forth. Searches can also be performed on previous search results and field tags can be combined using the Boolean operators "AND" and "OR". The operator "NOT" is only used in VIP.

### Schistosomiasis in the Chinese literature

#### Setting the stage: the global and Chinese contexts

Schistosomiasis remains one of the most prevalent parasitic diseases in the world with more than 200 million individuals infected, of whom over half suffer from related morbidity [14]. Whilst the global burden of schistosomiasis has been estimated at 1.7 to 4.5 million disability-adjusted life years [15,16], new research suggests this is a considerable underestimation of the 'true' burden of schistosomiasis [17,18]. However, schistosomiasis is a so-called neglected tropical disease, because it primarily affects poor rural communities in developing countries [19,20].

*Schistosomiasis japonica *has been known in China for over two millennia, but the first case report from China was not published until 1905 [21]. Towards the end of the 1940s, the central government of China recognized the intolerable burden of schistosomiasis and initiated a national control programme [11,12]. The third national sampling survey, completed in 2004, enrolled more than 250,000 individuals from 239 villages from the seven provinces where *Schistosoma japonicum *remains endemic. Human prevalence ranged from 0.3% in Jiangsu province to 4.2% in Hunan province. Extrapolating these data, the total number of infected individuals in China was estimated at 726,112 in 2004 [22]. This estimate translates to a reduction of 16.1% when compared with the previous national survey carried out in 1995, and dramatically lower than the 10 million Chinese estimated to be infected in the mid-1950s [11,12].

#### Searching Chinese biomedical databases

To illustrate how to search and make use of the Chinese scientific databases, we provide the following example. The keyword "*Schistosoma*" () was used to search the CNKI and VIP databases. Publication dates were set from 1990 to 2006. The results were imported into the reference management programme EndNote version 9 (Thomson ResearchSoft, Stamford, USA). The articles were stratified by journals, and sorted in descending order, i.e. number of articles published in the specified time frame. The journals were subdivided into core and non-core journals. The name, year when the journal was launched, language (abstract and main text) and open access status were recorded.

Additionally, we performed a content analysis and grouped the identified articles according to the following categories: (i) development, validation and application of new tools, (ii) epidemiology, (iii) control, and (iv) other schistosomiasis-related research. This analysis was stratified into two time periods: (i) 1990–1999 and (ii) 2000–2006.

#### Key Chinese journals for schistosomiasis research

**Figure 1:**
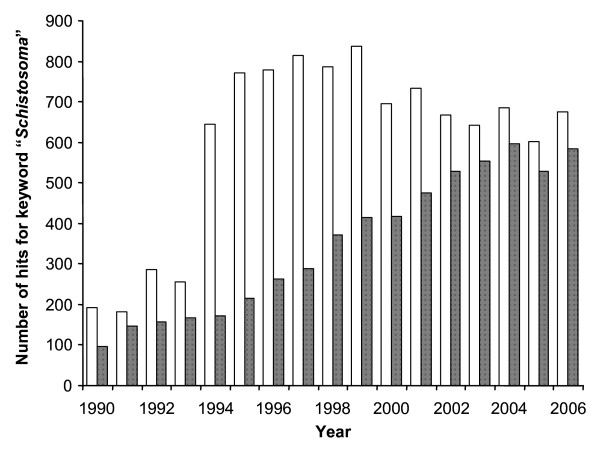
**Number of hits for the keyword "*****Schistosoma*****" (****) in two widely used Chinese databases: CNKI (white bars) and VIP (shaded bars) from 1990–2006.**

For the period 1990–2006, we obtained 10,244 hits in the CNKI and 5,975 hits in the VIP databases when using the keyword "*Schistosoma*" (). Figure 1 shows the number of hits per year for the two bibliographic databases. Three points are worth highlighting. First, more references were retrieved by the CNKI database compared with VIP in each year, but this gap has narrowed over time. Second, after a sharp increase in the number of hits on the CNKI database from 1993 (n = 255) to 1994 (n = 645), the annual number of hits in subsequent years remained relatively stable (602–838). Third, there was a gradual increase in the number of hits per year in the VIP database from 96 hits in 1990 to 585 hits in 2006.

In the 17-year period we examined, the 10 leading Chinese journals publishing original research on schistosomiasis were (i) *Chinese Journal of Schistosomiasis Control *(*Zhongguo Xue Xi Chong Bing Fang Zhi Za Zhi*), (ii) *Journal of Tropical Diseases and Parasitology *(*Re Dai Bing Yu Ji Sheng Chong Xue*), (iii)* Chinese Journal of Parasitology and Parasitic Diseases *(*Zhongguo Ji Sheng Chong Xue Yu Ji Sheng Chong Bing Za Zhi*), (iv)* Parasitoses and Infectious Diseases *(*Ji Sheng Chong Bing Yu Gan Ran Xing Ji Bing*, as well as its former title between 1994–2002, *Journal of Practical Parasitic Diseases*, *Shi Yong Ji Sheng Chong Bing Za Zhi*), (v)* Journal of Pathogen Biology *(*Zhongguo Bing Yuan Sheng Wu Xue Za Zhi*, as well as its former title between 1988–2005, *Chinese Journal of Parasitic Disease Control*, *Zhongguo Ji Sheng Chong Bing Fang Zhi Za Zhi*), (vi) *Chinese Journal of Zoonoses *(*Zhongguo Ren Shou Gong Huan Bing Xue Bao*, as well as its former title between 1985–2005, *Zhongguo Ren Shou Gong Huan Bing Za Zhi*), (vii) *International Journal of Medical Parasitic Diseases *(*Guo Ji Yi Xue Ji Sheng Chong Bing Za Zhi*, as well as its former title between 1994–2005, *Foreign Medicine Sciences Parasitic Diseases*, *Guo Wai Yi Xue Ji Sheng Chong Bing Fen Ce*), (viii) *Journal of Public Health and Preventive Medicine *(*Gong Gong Wei Sheng Yu Yu Fang Yi Xue*, as well as its former title between 1994–2004, *Hubei Journal of Preventive Medicine*, *Hubei Yu Fang Yi Xue Za Zhi*), (ix) *Practical Preventive Medicine *(*Shi Yong Yu Fang Yi Xue*), and (x) *China Tropical Medicine *(*Zhongguo Re Dai Yi Xue*). The cumulative numbers of *Schistosoma*-specific articles that were published by these journals were 2,954, 858, 674, 607, 458, 354, 262, 217, 180 and 82, respectively. Original research articles accounted for 70.8–90.8% of the total share of published work in nine of the 10 examined journals, whereas in the *International Journal of Medical Parasitic Diseases *the respective fraction was only 30.2%. *China Tropical Medicine *was launched in 2001, which partly explains its lower quantity of published papers. These 10 journals except the *International Journal of Medical Parasitic Diseases *and the *Journal of Public Health and Preventive Medicine *are all core journals and they comply with high academic standards. Submitted manuscripts, particularly original research and reviews, are usually peer-reviewed by at least two external referees. The *Chinese Journal of Parasitology and Parasitic Diseases*, which is hosted by the National Institute of Parasitic Diseases, Chinese Center for Disease Control and Prevention in Shanghai, for example, convenes six editorial meetings per year to come forward with final editorial decisions on which manuscripts to publish in the six issues per year. Articles usually contain Chinese and English summaries. Commencing in 2005, the *International Journal of Medical Parasitic Diseases *routinely publishes full-text English articles. The main body of some articles in *Chinese Journal of Parasitic Disease Control*, *Chinese Journal of Parasitology and Parasitic Diseases *and *China Tropical Medicine *are also published in English. The main body of articles published in the remaining six journals are usually only available in Chinese 1.

At present, only one of these 10 journals is indexed in PubMed under its Chinese name, *Zhongguo Ji Sheng Chong Xue Yu Ji Sheng Chong Bing Za Zhi *(*Chinese Journal of Parasitology and Parasitic Diseases*; indexed on PubMed since 1987). *China Tropical Medicine *is indexed on PubMed under its English name. Moreover, the following four journals are available from the 'Journals Database' in NCBI: (i) *Chinese Journal of Schistosomiasis Control *(*Zhongguo Xue Xi Chong Bing Fang Zhi Za Zhi*); (ii) *Chinese Journal of Parasitology and Parasitic Diseases *(*Zhongguo Ji Sheng Chong Xue Yu Ji Sheng Chong Bing Za Zhi*); (iii) *Journal of Pathogen Biology *(*Zhongguo Bing Yuan Sheng Wu Xue Za Zhi*) and (iv) *Chinese Journal of Zoonoses *(*Zhongguo Ren Shou Gong Huan Bing Xue Bao*). When searching PubMed for the term "*Schistosoma*" and restricting the search to the journal "*Zhongguo Ji Sheng Chong Xue Yu Ji Sheng Chong Bing Za Zhi*" and setting the temporal limits to 1990–2006, the total number of articles retrieved was 302, which translates to 44.8% of those found on the Chinese databases. None of the 10 Chinese journals examined here are yet included in the ISI Web of Knowledge. However, since the 1990s China has developed a similar citation database, namely the Chinese Science Citation Database, which contained already approximately 1,000 Chinese journals in 2001 [23]. With regard to open access, this feature was available for *China Tropical Medicine *in 2005 and 2006 via the website [24]. The *Chinese Journal of Parasitology and Parasitic Diseases *allowed open access sine 2003 directly on the journal website [25].

#### Content analysis

The retrieved *Schistosoma*-specific articles from the 10 leading Chinese journals were classified into four main groups, namely (i) new tools (e.g. development, validation and application of novel diagnostics, drugs and vaccines), (ii) epidemiology (e.g. epidemiological surveys, monitoring and surveillance), (iii) control (e.g. chemotherapy, health education, water supply and sanitation, and integrated control measures), and (iv) other research. Moreover, a temporal analysis was carried out, and we compared the relative frequencies of different research topics between the time periods 1990–1999 and 2000–2006.

Table 2 shows that in the 1990s, a total of 3,240 papers pertaining to different aspects of schistosomiasis were published in the 10 journals. One of the selected journals (*China Tropical Medicine*) was only launched in the new millennium, so the analysis of publications in the 1990s was based on nine rather than 10 journals. The development, validation and application of new tools and epidemiological research were the most important fields of endeavour, accounting for 26.8% and 29.5% of the original research articles published, respectively. With regard to new tools, diagnostics (11.9%) and drugs (9.7%) had a considerably higher share of the total publications than vaccines (5.2%). Publications with a focus on control accounted for 21.2%. The remaining publications (22.4%) pertained to other aspects of schistosomiasis research. Between journals, the proportions of each field of *Schistosoma*-related publications varied. For example, the *Chinese Journal of Zoonoses *published a large number of schistosomiasis- research articles that pertained to new tools (49.0%). *Parasitoses and Infectious Diseases *had the highest proportion of epidemiological research on schistosomiasis (45.2%). The two journals *Practical Preventive Medicine *and *Journal of Public Health and Preventive Medicine *had the highest share of articles dealing with schistosomiasis control: 39.2% and 38.6%, respectively.

**Table 2:** Total number of *Schistosoma*-specific articles published from 1990–1999 in the 10 leading Chinese journals publishing original schistosomiasis research articles in China, by major research topic

Journal	Total articles	New tools			Epidemiology	Control					Other research
					
		Diagnostics	Drugs	Vaccines		Clinical issues	Chemotherapy	Health education	Water supply and sanitation	Integrated control	
*Chinese Journal of Schistosomiasis Control*	1576	183 (11.6%)	152 (9.6%)	63 (4.0%)	502 (31.9%)	39 (2.5%)	146 (9.3%)	35 (2.2%)	17 (1.1%)	78 (4.9%)	361 (22.9%)
*Journal of Tropical Diseases and Parasitology*	520	56 (10.8%)	45 (8.6%)	28 (5.4%)	203 (39.0%)	14 (2.7%)	39 (7.5%)	16 (3.1%)	50 (9.6%)	38 (7.3%)	31 (6.0%)
*Chinese Journal of Parasitology and Parasitic Diseases*	320	29 (9.1%)	41 (12.8%)	3 (0.9%)	48 (15.0%)	7 (2.1%)	4 (1.3%)	3 (0.9%)	3 (0.9%)	4 (1.3%)	178 (55.6%)
*Parasitoses and Infectious Diseases*	292	51 (17.5%)	12 (4.1%)	3 (1.0%)	132 (45.2%)	18 (6.2%)	7 (2.4%)	16 (5.5%)	0	24 (8.2%)	29 (9.9%)
*Journal of Pathogen Biology*	166	15 (9.0%)	29 (17.5%)	17 (10.2%)	23 (13.9%)	4 (2.4%)	8 (4.8%)	3 (1.8%)	6 (3.6%)	4 (2.4%)	57 (34.3%)
*Chinese Journal of Zoonoses*	114	15 (13.1%)	14 (12.2%)	27 (23.7%)	2 (1.8%)	13 (11.4%)	3 (2.6%)	2 (1.8%)	2 (1.8%)	1 (0.9%)	35 (30.7%)
*Journal of Public Health and Preventive Medicine*	101	17 (16.8%)	11 (10.9%)	2 (2.0%)	23 (22.8%)	17 (16.8%)	6 (5.9%)	4 (4.0%)	2 (2.0%)	10 (9.9%)	9 (8.9%)
*Practical Preventive Medicine*	97	16 (16.5%)	7 (7.2%)	3 (3.1%)	22 (22.7%)	10 (10.3%)	10 (10.3%)	5 (5.2%)	2 (2.1%)	11 (11.3%)	11 (11.3%)
*International Journal of Medical Parasitic Diseases*	54	3 (5.6%)	4 (7.4%)	22 (40.7%)	2 (3.7%)	0	1 (1.9%)	0	0	5 (9.2%)	17 (31.5%)
*China Tropical Medicine*	n.a.	n.a.	n.a.	n.a.	n.a.	n.a.	n.a.	n.a.	n.a.	n.a.	n.a.

Total	3240	385 (11.9%)	315 (9.7%)	168 (5.2%)	957 (29.5%)	122 (3.8%)	224 (6.9%)	84 (2.6%)	82 (2.5%)	175 (5.4%)	728 (22.4%)

n.a. not applicable/not available

In the period 2000–2006, a total of 2,448 articles with an emphasis on schistosomiasis had been published in the 10 selected Chinese journals (Table 3). We found slightly higher proportions of published research pertaining to the development, validation and application of new tools (30.5%) and epidemiology (26.6%) when compared with the 1990s. On the other hand, the frequency of articles that focused on schistosomiasis control issues had not changed much (21.2% in the 1990s and 21.7% in 2000 – 2006). Generally, the proportion of articles on different aspects of schistosomiasis published by the individual journals were similar between the 1990s and the new millennium. Our results suggest that the journals examined here place particular emphasis on specific topics, and hence develop specific niches. *Parasitoses and Infectious Diseases*, for example, primarily covered epidemiological research on schistosomiasis (56.6%), whereas in the *Chinese Journal of Zoonoses *and *China Tropical Medicine*, more than half of the schistosomiasis-related articles focused on the development, validation and application of new tools.

**Table 3:** Total number of *Schistosoma*-specific articles published from 2000–2006 in the 10 leading Chinese journals publishing original schistosomiasis research articles in China, by major research topic

Journal	Total articles	New tools			Epidemiology	Control					Other research
					
		Diagnostics	Drugs	Vaccines		Clinical issues	Chemotherapy	Health education	Water supply and sanitation	Integrated control	
*Chinese Journal of Schistosomiasis Control*	1154	128 (11.1%)	134 (11.6%)	40 (3.5%)	382 (33.1%)	117 (10.1%)	28 (2.4%)	31 (2.7%)	16 (1.4%)	67 (5.8%)	211 (18.3%)
*Chinese Journal of Parasitology and Parasitic Diseases*	230	23 (10.0%)	21 (9.1%)	17 (7.4%)	33 (14.3%)	6 (2.6%)	0	0	0	5 (2.2%)	125 (54.3%)
*Parasitoses and Infectious Diseases*	205	28 (13.6%)	11 (5.4%)	2 (1.0%)	116 (56.6%)	12 (5.9%)	5 (2.4%)	16 (7.8%)	1 (0.5%)	10 (4.9%)	4 (2.0%)
*Journal of Tropical Diseases and Parasitology*	186	20 (10.8%)	17 (9.1%)	10 (5.4%)	69 (37.1%)	16 (8.6%)	5 (2.7%)	2 (1.1%)	12 (6.4%)	18 (9.6%)	17 (9.1%)
*Chinese Journal of Parasitic Disease Control*	179	12 (6.7%)	20 (11.1%)	36 (20.1%)	30 (16.8%)	14 (7.8%)	2 (1.1%)	5 (2.8%)	4 (2.2%)	9 (5.0%)	47 (26.3%)
*Chinese Journal of Zoonoses*	156	16 (10.3%)	7 (4.5%)	68 (43.6%)	10 (6.4%)	3 (1.9%)	1 (0.6%)	0	0	3 (1.9%)	48 (30.8%)
*International Journal of Medical Parasitic Diseases*	141	15 (10.6%)	13 (9.2%)	35 (24.8%)	15 (10.6%)	3 (2.1%)	0	0	2 (1.4%)	8 (5.7%)	50 (35.5%)
*Journal of Public Health and Preventive Medicine*	71	12 (16.9%)	5 (7.0%)	2 (2.8%)	21 (29.6%)	3 (4.2%)	4 (5.6%)	2 (2.8%)	3 (4.2%)	16 (22.5%)	3 (4.2%)
*China Tropical Medicine*	67	2 (3.0%)	8 (11.9%)	25 (37.3%)	15 (22.4%)	3 (4.5%)	0	1 (1.5%)	3 (4.5%)	3 (4.5%)	7 (10.4%)
*Practical Preventive Medicine*	59	9 (15.2%)	6 (10.2%)	5 (8.5%)	14 (23.7%)	8 (13.6%)	6 (10.2%)	0	1 (1.7%)	6 (10.2%)	4 (6.8%)

Total	2448	265 (10.8%)	242 (9.9%)	240 (9.8%)	652 (26.6%)	238 (9.7%)	51 (2.1%)	57 (2.3%)	42 (1.7%)	145 (5.9%)	516 (21.1%)

### Harnessing the Chinese literature

#### Discovery, development and use of antischistosomal drugs

A total of 328 articles were retrieved in the CNKI database when we searched for publications pertaining to the discovery, development and use of antischistosomal drugs. A significant amount of research focused on praziquantel, an antischistosomal drug discovered in the mid-1970s by Bayer in Germany [26,27]. The first clinical use of praziquantel in China dates back to the late 1970s [28]. Since then, praziquantel has become the drug of choice for the control of schistosomiasis worldwide [15,29]. More than 50 million treatment courses have been administered in China and comprehensive reviews have recognised the significant contributions from Chinese clinicians and scientists towards the optimization of praziquantel use for individual treatment and community-based morbidity control [30,31]. The large-scale deployment of praziquantel, particularly during the 10-year World Bank Loan Project (WBLP) (1992–2001) to control schistosomiasis in China, significantly reduced the morbidity due to *S. japonicum *[32]. To date, there is no clear evidence of clinically relevant resistance to praziquantel and there are basically no alternative drugs available, although perhaps praziquantel analogues could be developed. An important limitation of praziquantel is that it lacks activity against the young developing stages of *S. japonicum *[33].

In 1980, Chinese scientists discovered that artemisinin, in addition to its antimalarial activity, also exhibited antischistosomal properties [34]. Studies suggested that artemisinin and its derivatives (artemether and artesunate) could kill the early developmental stages of the parasite, thus preventing the development of egg-laying adult worms, and hence preventing the associated morbidity from the immunological reactions to schistosome eggs trapped in the host tissue. In 2000, the key findings of 20 years of laboratory research and the evidence generated from clinical trials were published in English in one of the leading international parasitology journals [35]. Subsequently, the data were also reviewed and published in the *Chinese Journal of Schistosomiasis Control*, of course in Chinese [36]. In view of the artemisinins exhibiting highest activity against juvenile *S. japonicum *worms, ensuing research was aimed at developing the artemisinins as chemoprophylactic agents against schistosomiasis. Early administration of artemether was highly efficacious in preventing patent infections among flood relief workers exposed to schistosome-infested water [37]. Importantly, this body of research stimulated scientists outside China to assess the effect of the artemisinins on the other two major human schistosome species, i.e. *S. haematobium *and *S. mansoni*, first in the laboratory, followed by clinical trials (for a recent review see [38]).

#### Molluscicides and environmental management

From the CNKI database, a total of 290 articles pertaining to molluscicides and environmental management for schistosomiasis control were retrieved. Different approaches regarding the possible elimination of the intermediate host snail (genus: *Oncomelania*) have been reported, including biological methods (e.g. introduction of competitor snails), chemical control (e.g. use of molluscicides) and environmental management. With regard to molluscicides, niclosamide is the most widely used product in China [39-41]. A number of new compounds have been developed in China and their molluscicidal properties evaluated in the laboratory and in small pilot studies but, thus far, these compounds have not been applied on a wider scale in the field. The compounds include bromoacetamide, nicotinanilide, metaldehyde, thiocyclohexane oxalate and l,3-bis-sodium thiosulfate-2-dimethylaminopropane [42-44]. One of the compounds, namely 40% META liquid (a metaldehyde product licensed by Lonza Ltd., Basel, Switzerland) shows a promising molluscicidal effect against *Oncomelania *with low toxicity to fish. Hence this compound holds particular promise for the control of intermediate host snails, and thus warrants further investigation [45]. Moreover, some compounds impede the snail's sensory system and permit an increased area of softbody exposure, thus enhancing molluscicidal activities when combined with other agents [46]. Therefore, several new molluscicide formulations, including powder formulations and suspension concentrate formulations were developed to increase the efficacy of niclosamide.

Chinese researchers have also tested plant extracts from *Arca catectu*, *Camellia oleosa*, *Eucalyptus camaldulensis*, *Jatropha curcas*, *Leonurus artemisia*, *Rhododendron sinensis*, among others, for their molluscicidal properties [47-49]. Most of these plant extracts showed potential effect against *Oncomelania*, but due to low yields extracted from raw plant material, it is unlikely that these compounds will play an important role for mollusciciding programmes in the near future unless the active ingredient can be isolated and synthesized [48,49].

A considerable number of articles focused on environmental management and their role for enhancing the national schistosomiasis control programme in China. Prominent examples are integration of environmental management with agriculture activities, e.g. alteration of crops planted, reclamation of land and plantations to eliminate snail habitats [50]. Integration of environmental management with water resources development projects has also been carried out, e.g. cement lining of irrigation canals, installation of sinks to obstruct snail dispersal, and construction of flood gates in the connection areas of main irrigation canals or rivers. Integration of environmental management with forestry projects is another example, e.g. planting trees in the marshlands to change the ecology of snail habitats and to discourage buffaloes from grazing on the marshland, and establishing new forests in the mountainous areas [51,52]. Finally, integration of environmental management with agricultural projects has also been implemented, e.g. construction of fish ponds, rearrangement of irrigation systems and establishment of drainage systems.

In the lake regions of Anhui, Hubei, Hunan, Jiangsu and Jiangxi provinces, a diversity of ecological and environmental measures were employed and their effect on reducing or even eliminating the intermediate host snails and interrupting the transmission of *S. japonicum *was assessed. The interventions consisted of (i) constructing fish ponds, (ii) digging new ditches and filling of infested ones, (iii) reclaiming land and planting trees, (iv) earth burying, (v) concreting over ditches and (vi) combining different environmental management interventions [53,54]. The results of these investigations suggested that environmental management provides effective measures not only for in reducing or even eliminating snail habitats, but also for in markedly reducing the prevalence of human schistosomiasis. Comprehensive agricultural development and environment modifications as described, in conjunction with chemotherapy targeting both humans and domestic animals, demonstrated success in significantly reducing the prevalence of human schistosomiasis in China [50-53]. In particular, new hydraulic technologies for preventing the spread of *Oncomelania *during flooding have been explored and integrated into the local control programmes in the lake region, based on an enhanced understanding of snail ecology and hydrology [51,55-59].

In the mountainous areas of Sichuan and Yunnan provinces, environmental control measures such as constructing reservoirs, and modifying the complex environment showed some success in reducing the number of intermediate host snails and lowering the human infection prevalence. However, there is considerable concern that the damming of the Yangtze River, particularly due to the Three Gorges Dam, will lead to the emergence or re-emergence of schistosomiasis [54]. This once again calls for rigorous monitoring and surveillance.

The development of special types of latrines with three sinks for the treatment of 'night-soil' and the production of biogas, as well as issuing new national guidelines for drinking water supply are also worth noting. Indeed, Chinese research has shown that setting aside human faecal matter intended for 'night-soil' for at least 15 days reduced miracidia hatching of schistosome eggs by up to 99.8% [50].

### China's influence on local schistosomiasis control strategies and global policy

China established a national leading group for schistosomiasis control at the central government level early on. From the second half of the 1950s onwards, leading groups were established at different administrative levels, e.g. at provincial, county and township levels. Members of these leading groups were drawn from the health, agriculture and the water sectors, and hence intersectoral collaboration was fostered. For the implementation of schistosomiasis control measures – with an initial focus on snail control – large numbers of local residents were mobilized to carry out these tasks [59,60].

Numerous provincial and county anti-schistosomiasis control stations have been created in China. In the 1980s, for example, approximately 16,000 professionals were responsible for controlling schistosomiasis across the country. The policy at the time emphasised prevention and aimed at a comprehensive control approach, readily adapted to the different eco-epidemiological settings. Hence, multiple control measures were implemented simultaneously and the ultimate objective was to interrupt transmission in an efficient and cost-effective manner [55].

In the mid-1980s, an expert committee of the World Health Organization (WHO) endorsed a new schistosomiasis control strategy that shifted the focus from transmission control to morbidity control. Simply put, it emphasised that people, rather than snails, cause schistosomiasis. By adopting the new strategy, more emphasis was placed on health education, large-scale screening and chemotherapy, access to clean water and improved sanitation. In China, however, snail control was never abandoned and intersectoral collaboration between health, education, and agriculture and water authorities remained a key feature of the national schistosomiasis control programme [11,52]. The central role that China placed on health education to reduce the risk of human transmission is reflected in the number of people involved; China employed 40,000 staff in health education for schistosomiasis control in 1995 [58]. Information, education and communication materials were developed and validated, and key messages for the prevention of schistosomiasis were broadcasted through China's mass media. In the 1990s more than 2 million textbooks for the prevention and control of schistosomiasis were distributed in primary and secondary schools in China's schistosome-endemic areas. Monitoring of these activities revealed that the coverage rate of health education for schistosomiasis control among schoolchildren approached 100% [56,57].

The last 15 years of schistosomiasis control in China can be summarized as follows. First, the objectives of the control programme gradually shifted from morbidity control to transmission control and, finally, transmission interruption [58-60]. The WBLP funds for schistosomiasis control, which ran from 1992–2001, were an important component of the "Eighth Five-Year Plan" (1991–1995) and the "Ninth Five-Year Plan" (1996–2000) of China's national schistosomiasis control programme [32]. The WBLP emphasised morbidity control using praziquantel, and continued to adopt disease surveillance, health education and applied research [52]. In 1998, an important document entitled "A plan of action on surveillance and consolidation for schistosomiasis in transmission interrupted areas" was produced by the Chinese health authorities [61]. In 2004, "An outline of the mid-term and long-term programme for schistosomiasis control and prevention in China" was issued by the State Council, and in 2006, the "National Regulation of Schistosomiasis Control" was published by the State Department of China [62].

Medical breakthroughs developed first in China, as exemplified in the case of the artemisinins against malaria and schistosomiasis [38], have made a huge impact in the research community and on global health. China has a track record of developing and implementing truly integrated control measures against schistosomiasis, but much of China's decades of knowledge have yet to become fully accessible and be realised outside of the country.

## Conclusion

China's social and economic advancement, its bountiful talent and the embrace of new technologies have all fuelled scientific research activities along with the development of important biomedical bibliographic databases. The potential of the Chinese literature to advance biomedical research has been stressed more than a decade ago [63], but challenges for the Chinese journals have also been highlighted [64,65]. As our example in schistosomiasis research illustrates, the number of *Schistosoma*-related articles alone from China increased from 193 in 1990 to 676 in 2006 in the CNKI database and from 96 to 585 in the VIP database over the same time period. From these two databases we retrieved and analysed more than 3,000 articles focusing on original schistosomiasis research and determined the leading 10 Chinese journals in the field of schistosomiasis research. Most of the articles were published in Chinese, often with an English summary.

We have highlighted significant contributions from the Chinese literature, exemplified by two key areas of schistosomiasis research: (i) drug discovery, and (ii) molluscicides and environmental management for integrated control approaches. Research from China has enhanced our antischistosomal drug and molluscicidal armamentarium and the understanding of the local epidemiology and control of schistosomiasis. We have used one area of research as an example to illustrate how the Chinese scientific literature can influence global health and research. Unfortunately, there are often long delays in the international dissemination of important knowledge from non-English speaking countries such as China to the wider research community. Concerted efforts should be made and innovative methods developed to promote open, rapid and accurate access to new knowledge and to overcome language barriers.

## Abstracts in non-English languages

The abstract of this paper has been translated into the following languages by the following translators (names in brackets):

• Chinese – simplified characters (Dr. Qin Liu and Prof. Xiao-Nong Zhou) [see Additional file 1]

• Chinese – traditional characters (Dr. Qin Liu and Prof. Xiao-Nong Zhou) [see Additional file 2]

• French (Mr. Philip Harding-Esch) [see Additional file 3]

• German (Dr. Peter Steinmann) [see Additional file 4]

• Spanish (Ms. Annick Borquez) [see Additional file 5]

## Additional Table 1

Characteristics of the 10 Chinese journals publishing the highest number of original schistosomiasis research articles were summarized in Additional Table 1. [see Additional file 6]

## Competing interests

The authors declare that they have no competing interests.

## Authors' contributions

QL retrieved the data from the Chinese biomedical databases, carried out the quantitative analysis and content analysis and contributed to drafting the manuscript. LGT assisted with the data retrieval and analysis of the data. SHX interpreted the data on drug discovery and development and drafted this part of the manuscript. ZQ assisted in the data retrieval and analysis of the data. PS assisted in the interpretation of the data and the drafting and revision of the manuscript. TKM contributed to the idea of this manuscript and revised the manuscript. JU conceived the manuscript, checked and interpreted the data, drafted and revised the manuscript. XNZ conceived the manuscript, checked and interpreted the data, drafted and revised the manuscript, and is the guarantor of the paper. All authors read and approved the final manuscript.

## Supplementary Material

Additional File 1 Abstract in Chinese – Simplified characters. Click here for file

Additional File 2 Abstract in Chinese – traditional characters. Click here for file

Additional File 3 Abstract in French. Click here for file

Additional File 4 Abstract in German. Click here for file

Additional File 5 Abstract in Spanish. Click here for file

Additional File 6 Additional Table 1. Characteristics of the 10 Chinese journals publishing the highest number of original schistosomiasis research articles. Click here for file

## References

[B1] Fung ICH (2008). Chinese journals: a guide for epidemiologists. Emerg Themes Epidemiol.

[B2] Xia J, Wright J, Adams CE (2008). Five large Chinese biomedical bibliographic databases: accessibility and coverage. Health Info Libr J.

[B3] Li Y, Wu YL (2003). An over four millennium story behind qinghaosu (artemisinin) – a fantastic antimalarial drug from a traditional Chinese herb. Curr Med Chem.

[B4] Wang MW, Hao XJ, Chen KX (2007). Biological screening of natural products and drug innovation in China. Philos Trans R Soc Lond B Biol Sci.

[B5] China Cooperative Research Group on Qinghaosu and its Derivatives as Antimalarials (1982). Studies on the toxicity of qinghaosu and its derivatives. J Tradit Chin Med.

[B6] China Cooperative Research Group on Qinghaosu and its Derivatives as Antimalarials (1982). Chemical studies on qinghaosu (artemisinine). J Tradit Chin Med.

[B7] China Cooperative Research Group on Qinghaosu and its Derivativesas Antimalarials (1982). Antimalarial efficacy and mode of action of qinghaosu and its derivatives in experimental models. J Tradit Chin Med.

[B8] China Cooperative Research Group on Qinghaosu and its Derivatives as Antimalarials (1982). Clinical studies on the treatment of malaria with qinghaosu and its derivatives. J Tradit Chin Med.

[B9] Klayman DL (1985). Qinghaosu (artemisinin): an antimalarial drug from China. Science.

[B10] Haynes RK (2006). From artemisinin to new artemisinin antimalarials: biosynthesis, extraction, old and new derivatives, stereochemistry and medicinal chemistry requirements. Curr Top Med Chem.

[B11] Utzinger J, Zhou XN, Chen MG, Bergquist R (2005). Conquering schistosomiasis in China: the long march. Acta Trop.

[B12] Zhou XN, Wang LY, Chen MG, Wu XH, Jiang QW, Chen XY, Zheng J, Utzinger J (2005). The public health significance and control of schistosomiasis in China – then and now. Acta Trop.

[B13] Fenwick A, Rollinson D, Southgate V (2006). Implementation of human schistosomiasis control: challenges and prospects. Adv Parasitol.

[B14] Steinmann P, Keiser J, Bos R, Tanner M, Utzinger J (2006). Schistosomiasis and water resources development: systematic review, meta-analysis, and estimates of people at risk. Lancet Infect Dis.

[B15] WHO (2002). Prevention and control of schistosomiasis and soil-transmitted helminthiasis: report of a WHO expert committee. World Health Organ Tech Rep Ser.

[B16] WHO (2004). The world health report 2004: changing history.

[B17] King CH, Dickman K, Tisch DJ (2005). Reassessment of the cost of chronic helmintic infection: a meta-analysis of disability-related outcomes in endemic schistosomiasis. Lancet.

[B18] Jia TW, Zhou XN, Wang XH, Utzinger J, Steinmann P, Wu XH (2007). Assessment of the age-specific disability weight of chronic schistosomiasis japonica. Bull World Health Organ.

[B19] Hotez PJ, Molyneux DH, Fenwick A, Kumaresan J, Ehrlich Sachs S, Sachs JD, Savioli L (2007). Control of neglected tropical diseases. N Engl J Med.

[B20] Keiser J, Utzinger J (2007). Advances in the discovery and development of novel trematocidal drugs. Expert Opin Drug Discov.

[B21] Logan OT (1905). A case of dysentery in Hunan province caused by the trematode *Schistosoma japonicum*. China Med Missionary J.

[B22] Zhou XN, Guo JG, Wu XH, Jiang QW, Zheng J, Dang H, Wang XH, Xu J, Zhu HQ, Wu GL (2007). Epidemiology of schistosomiasis in the People Republic of China, 2004. Emerg Infect Dis.

[B23] Leydesdorff L, Bihui J (2005). Mapping the Chinese Science Citation Database in terms of aggregated journal-journal citation relations. J Am Soc Inf Sci Technol.

[B24] China Tropical Medicine. http://journal.shouxi.net/qikan/guokanll_zzxq.php?id=121.

[B25] Chinese Journal of Parasitology and ParasiticDiseases. http://www.jsczz.cn/cn/dqml.asp.

[B26] Seubert J, Pohlke R, Loebich F (1977). Synthesis and properties of praziquantel, a novel broad spectrum anthelmintic with excellent activity against schistosomes and cestodes. Experientia.

[B27] Groll E (1984). Praziquantel. Adv Pharmacol Chemother.

[B28] Yang Y, Shen GR, Mei YS, Zhang CH, Ying LZ, You BY, Dong YQ, Fu S, Wu HM, Qie YD (1981). Study on the new drug praziquantel for schistosomiasis. New Med.

[B29] Utzinger J, Keiser J (2004). Schistosomiasis and soil-transmitted helminthiasis: common drugs for treatment and control. Expert Opin Pharmacother.

[B30] Chen MG (2005). Use of praziquantel for clinical treatment and morbidity control of schistosomiasis japonica in China: a review of 30 years' experience. Acta Trop.

[B31] Xiao SH (2005). Development of antischistosomal drugs in China, with particular consideration to praziquantel and the artemisinins. Acta Trop.

[B32] Chen XY, Wang LY, Cai JM, Zhou XN, Zheng J, Guo JG, Wu XH, Engels D, Chen MG (2005). Schistosomiasis control in China: the impact of a 10-year World Bank Loan Project (1992-2001). Bull World Health Organ.

[B33] Xiao SH, Yue WJ, Yang YQ, You JQ (1987). Susceptibility of *Schistosoma japonicum *to different developmental stages to praziquantel. Chin Med J.

[B34] Chen DJ, Fu LF, Shao PP, Wu FZ, Fan CZ, Shu H, Ren CX, Sheng XL (1980). Experimental studies on antischistosomal activity of qinghaosu. Zhong Hui Yi Xue Zha Zhi.

[B35] Xiao SH, Booth M, Tanner M (2000). The prophylactic effects of artemether against *Schistosoma japonicum *infections. Parasitol Today.

[B36] Xiao SH (2005). Study on the prevention and cure efficacy of artemether against schistosomiasis. Chin J Schisto Cont.

[B37] Song Y, Xiao SH, Wu W, Zhang S, Xie HQ, Xu XP, Hu XY, Cui Q, Chen MG, Zheng J (1998). Preventive effect of artemether on schistosome infection. Chin Med J.

[B38] Utzinger J, Xiao SH, Tanner M, Keiser J (2007). Artemisinins for schistosomiasis and beyond. Curr Opin Investig Drugs.

[B39] Dai JR, Zhou XN, Liang YS, Zhang YP, Jiang YJ, Xi WP, Huang YX, Chen C, Huang MX, Zhu YC (2002). Sensitivity of *Oncomelania *snails to niclosamide in China. Zhongguo Ji Sheng Chong Xue Yu Ji Sheng Chong Bing Za Zhi.

[B40] Li HJ, Liang YS, Dai JR, Xu YL, Tang JX, Ru WW, Shen XH, Xu M, Zhu YC (2005). 25% concentrate suspension of niclosamide; *Schistosoma japonicum*; cercariae; effective concentration. Chin J Schisto Cont.

[B41] Wang ZC (2006). Cost-effectiveness analysis of eliminating *Oncomelania *with 4% niclosamide ethanolamine salt. J Public Health Prev Med.

[B42] Chen ZP, Tao HQ, Hua DS, Shen BR, Chan HL (1991). Evaluation of molluscicidal effect of nicotinanilide against *Oncomelania *snail. Zhongguo Ji Sheng Chong Xue Yu Ji Sheng Chong Bing Za Zhi.

[B43] Wu F, Chen YT, Dai JR, Gao ZH, Cao Q (1993). The molluscicidal effect of bromoacetamide on *Oncomelania *snails. Chin J Schisto Cont.

[B44] Wu F, Jiang YJ, Xi WP, Hong QB (2004). Study on the long-term molluscicidal effect of magnesium powder. Chin J Parasit Dis Cont.

[B45] Zhu D, Zhou XN, Zhang SQ, Zhang GH, Liu HX, Lu DB, Cai GY, Ni QZ, Cao ZG, Wu WD (2006). Study on the molluscicidal effect of META-Li against *Oncomelania hupensis*. Zhongguo Ji Sheng Chong Xue Yu Ji Sheng Chong Bing Za Zhi.

[B46] Xu XJ, Cai SX, Wei FH, Liu JB, Fu Y, Cao MM (2003). Study on increasing molluscicidal effect by complex nicotinanilide with niclosamide. Chin J Schisto Cont.

[B47] Hong QB, Zhou XN, Han Y, P. SL, Yang GJ (2001). Molluscicidal effects of extracts of *Eucalyptus camaldulensis *on *Oncomelania hupensis*. Chin J Schisto Cont.

[B48] Yang Z, Yin GL, Fan CZ, Wang SW, Luo TP, Liu YH, Duan YC, Chen JX, Li YL (2003). Molluscicidal effects of extracts of *Jatropha curcas *seeds on *Oncomelania hupensis*. Chin J Schisto Cont.

[B49] Liao BR, Wang WX, Zhang JL, Zhang Y, Hou JH, Shu LH (2006). Studies on the molluscicidal activity of *Leonurus artemisia *to *Oncomelania hupensis*. Biotechnol Bull.

[B50] Zuo JZ, Ren MY, He HB, Gao JX, Huang CL, Lin B, Hu SG (1999). Control of schistosomiasis in lake regions through snail control and environmental measures. Chin J Schisto Cont.

[B51] Gao LL (2002). Considerations on snail control by environmental modification in Zhejiang province. Chin J Schisto Cont.

[B52] Wang JG, Liao HY, Zhong GT, Yu BG, Huang SS (2002). Observation on the effect on schistosomiasis through snail control by environmental modification. Chin J Schisto Cont.

[B53] Zhang YQ, Zhang J, Shu RR, He QX, Qi LJ, Zhang R, Hu HJ, Shun WS, Yang H (2003). Effect of schistosomiasis control by environment alteration and chemotherapy in lake regions. Chin J Schisto Cont.

[B54] Li YS, Raso G, Zhao ZY, He YK, Ellis MK, McManus DP (2007). Large water management projects and schistosomiasis control, Dongting Lake region, China. Emerg Infect Dis.

[B55] Jia DY (1993). The epidemiology and control strategy of schistosome recently in China. Chin J Cont Endem Dis.

[B56] Yao JM, Su YX, Su ZD, Deng JM, Zeng LD, Li CM (1995). The importance of health education for schistosomiasis control. Chin J Schisto Cont.

[B57] Liu HX, Yu FG, Yang XQ (1998). The role of health education to improve schistosomiasis screening in human. Pract Prev Med.

[B58] Zhen J (2004). The challenge and development aim of schistosomiasis control in China. J Trop Dis Parasitol.

[B59] Zhen J (2006). Schistosomiasis control and its prospect in China. Chin J Schisto Cont.

[B60] Lin DD, Wu HW, Wu GL, Zhou XN (2007). Review and evaluation on optimal combined strategies for schistosomiasis control in China. Chin J Schisto Cont.

[B61] Zhou XN, Jiang QW, Shun LM, Wang TP, Hong QB, Zhao GM, Lin DD (2005). Schistosomiasis control and surveillance in China. Chin J Schisto Cont.

[B62] Wang LD (2006). Carry out ordinance, change the policy of schistosomiasis control. Chin J Prev Med.

[B63] Smith R (1994). Chinese medical journals: getting in touch. BMJ.

[B64] Gastel B, Weng YQ (1990). Medical journals in China. Ann Intern Med.

[B65] Ren S, Liang P, Zu G (1999). The challenge for Chinese scientific journals. Science.

