# Supplementary material for: Harnessing the wealth of Chinese scientific literature: schistosomiasis research and control in China
Source: Emerg Themes Epidemiol. 2008 Sep 30;5:19. doi: 10.1186/1742-7622-5-19 (PMC2576166; doi:10.1186/1742-7622-5-19)
Supplement: Additional File 1 — Abstract in Chinese – Simplified characters. [file 1742-7622-5-19-S1.pdf]

Simplified Chinese / 简体中文

分析透视

中国科学文献宝库的利用：中国血吸虫病研究与防治

作者：

刘琴（Qin Liu），田利光（Li-Guang Tian），肖树华（Shu-Hua Xiao），郑琪（Zhen Qi），彼得·斯大曼（Peter Steinmann），愸毕·马克（Tippi Mak），云格·乌辛格（Jürg Utzinger），周晓农（Xiao-Nong Zhou）

摘要：

与中国经济持续繁荣同步，生物医学研究与相应的文献发表也在增多。在发展中国家流行普遍的“被忽略的热带疾病”仍流行或重新流行于中国的部分地区。本文的目的是以中国血吸虫流行病学和控制研究为例，表明中国生物医学数据库的重要科研潜力。我们搜索了两大数据库，即中国知识基础设施（CNKI）和维普资讯（VIP），搜索使用关键词为“血吸虫”，时间期限为 1990-2006 年。在 CNKI 共搜索到 10,244 篇文章，在 VIP 共搜索到 5,975 篇文章。统计发表血吸虫病论著最多的 10 本生物医学期刊，包括了出版语言及是否免费获取等内容，发现大部分期刊都以中文发表，并通常同时提供英文摘要。在

这些期刊中，只有《中国热带医学》2005-2006 年的部分文章以及《中国寄生虫学与寄生虫病杂志》2003 年以后的文章可免费获取。我们从以下三方面综述了过去 20 年中国血吸虫病研究成果：（1）抗血吸虫药的研发进展；（2）灭螺药的研究进展；（3）血吸虫病防治中的环境治理研究。总之，中文文献中发表了很多重要的科研成果，既涉及了当地的防控策略，也有全球的科学知识。今后应鼓励免费开放文献，同时消除语言障碍，使中国的科研被全球科学界所认识。

译者：

刘琴（Qin Liu），周晓农（Xiao-Nong Zhou）
