# Supplementary material for: Harnessing the wealth of Chinese scientific literature: schistosomiasis research and control in China
Source: Emerg Themes Epidemiol. 2008 Sep 30;5:19. doi: 10.1186/1742-7622-5-19 (PMC2576166; doi:10.1186/1742-7622-5-19)
Supplement: Additional File 2 — Abstract in Chinese – traditional characters. [file 1742-7622-5-19-S2.pdf]

Traditional Chinese／ 繁體中文

分析透視

中國科學文獻寶庫的利用：中國血吸蟲病研究與防治

作者：

劉琴（Qin Liu）,田利光（Li-Guang Tian）,肖樹華（Shu-Hua Xiao）, 鄭琪（Zhen Qi）, 彼得•斯大曼（Peter Steinmann）, 悐畢•馬克（Tippi Mak）, 雲格•烏辛格（Jürg Utzinger）, 周曉農（Xiao-Nong Zhou）

摘要：

與中國經濟持續繁榮同步，生物醫學研究與相應的文獻發表也在增多。在發展中國家流行普遍的「被忽略的熱帶疾病」仍流行或重新流行於中國的部分地區。本文的目的是以中國血吸蟲流行病學和控制研究爲例，表明中國生物醫學數據庫的重要科研潛力。我們搜索了兩大數據庫，即中國知識基礎設施（CNKI）和維普資訊（VIP），搜索使用關鍵詞爲「血吸蟲」，時間期限爲 1990-2006 年。在 CNKI 共搜索到 10,244 篇文章，在 VIP 共搜索到 5,975 篇文章。統計發表血吸蟲病論著最多的 10 本生物醫學期刊，包括了出版語言及是否免費獲取等內容，發現大部分期刊都以中文發表，並通常同時提供英文摘

要。在這些期刊中，只有《中國熱帶醫學》2005-2006 年的部分文章以及《中國寄生蟲學與寄生蟲病雜誌》2003 年以後的文章可免費獲取。我們從以下三方面綜述了過去 20 年中國血吸蟲病研究成果：(1) 抗血吸蟲藥的研發進展；(2) 滅螺藥的研究進展；(3) 血吸蟲病防治中的環境治理研究。總之，中文文獻中發表了很多重要的科研成果，既涉及了當地的防控策略，也有全球的科學知識。今後應鼓勵免費開放文獻，同時消除語言障礙，使中國的科研被全球科學界所認識。

譯者：

劉琴（Qin Liu），周曉農（Xiao-Nong Zhou）
