# Supplementary material for: Harnessing the wealth of Chinese scientific literature: schistosomiasis research and control in China
Source: Emerg Themes Epidemiol. 2008 Sep 30;5:19. doi: 10.1186/1742-7622-5-19 (PMC2576166; doi:10.1186/1742-7622-5-19)
Supplement: Additional File 3 — Abstract in French. [file 1742-7622-5-19-S3.pdf]

French / Français

Perspectives analytiques

Exploiter la richesse de la littérature scientifique chinoise: la recherche sur la schistosomiase et son contrôle en Chine.

Auteurs: Qin Liu, Li-Guang Tian, Shu-Hua Xiao, Zhen Qi, Peter Steinmann, Tippi Mak, Jürg Utzinger, Xiao-Nong Zhou

Résumé

L’économie chinoise est toujours en plein essor et il en est de même pour la recherche biomédicale chinoise et les publications liées à cette recherche. Les maladies tropicales dites négligées, qui sont plus répandues dans les pays en voie de développement, sont toujours omniprésentes ou même émergentes dans certaines régions de Chine. L’objectif de cet article est de décrire le potentiel considérable que constituent les bases de données bibliographiques biomédicales chinoises pour la recherche. Les contributions chinoises à la recherche sur l’épidémiologie de la schistosomiase et son contrôle en sont une excellent illustration. Deux bases de données de grand usage ont été interrogées: la *China National Knowledge Infrastructure* (CNKI) et *VIP Information* (VIP). En utilisant le mot-clé « Schistosoma » (血吸虫) et couvrant la période 1990-2006, 10244 réponses pertinentes ont été obtenues avec la base de données CNKI et 5975 avec VIP. 10 revues biomédicales chinoises

qui ont publié le plus grand nombre d’articles de recherche sur la schistosomiase ont été examinées sous certains critères dont la langue et le libre accès (Open Access). Bien que la plupart des revues soient publiées en chinois, des résumés en anglais sont normalement disponibles. Le libre accès au texte intégral des articles était disponible pour la revue *China Tropical Medicine* dès 2005/2006 et il est permis par le *Chinese Journal of Parasitology and Parasitic Diseases* depuis 2003. Aucun des autres journaux étudiés ne permettait le libre accès. Nous avons considéré (i) la découverte et le développement des médicaments antibilharziens, (ii) les progrès en molluscicides et (iii) les progrès en matière de gestion environnementale pour le contrôle de la schistosomiase en Chine dans les 20 dernières années. Pour conclure, d’importantes études sont publiées dans la littérature scientifique chinoise, ces dernières sont pertinentes pour les mesures de contrôle local et pour le savoir scientifique mondial. Le libre accès devrait être encouragé et les barrières linguistiques éliminées afin de permettre à la communauté scientifique de mieux profiter de toute la richesse de la recherche chinoise.

(Traduit en français par Philip Harding-Esch)
