# Supplementary material for: Harnessing the wealth of Chinese scientific literature: schistosomiasis research and control in China
Source: Emerg Themes Epidemiol. 2008 Sep 30;5:19. doi: 10.1186/1742-7622-5-19 (PMC2576166; doi:10.1186/1742-7622-5-19)
Supplement: Additional File 4 — Abstract in German. [file 1742-7622-5-19-S4.pdf]

German / Deutsch

Analytische Perspektive

Den Reichtum der chinesischen Fachliteratur nutzen: Erforschung und Kontrolle der Schistosomiasis in China

Autoren: Qin Liu, Li-Guang Tian, Shu-Hua Xiao, Zhen Qi, Peter Steinmann, Tippi K. Mak, Jürg Utzinger, Xiao-Nong Zhou

Zusammenfassung

Die Wirtschaft Chinas wächst weiterhin schnell und eine vergleichbare Entwicklung zeigen die chinesische biomedizinische Forschung und die damit verbundenen Publikationsaktivitäten. Gewisse sogenannte „vernachlässigte Tropenkrankheiten" mit einem Verbreitungsschwerpunkt in Entwicklungsländern sind in Teilen Chinas nach wie vor weit verbreitet und teilweise sogar am Zunehmen. Das Ziel dieses Artikels ist es, das beachtliche Forschungspotenzial von chinesischen biomedizinischen Bibliographie-Datenbanken aufzuzeigen. Die Forschungsbeiträge aus China zur Epidemiologie und Kontrolle der Schistosomiasis demonstrieren dies auf eindrucksvolle Weise. Wir fragten zwei populäre Datenbanken ab, die „Nationale Wissensinfrastruktur Chinas" (CNKI) und „VIP Information" (VIP). Die Suche nach dem Begriff „*Schistosoma*" (血吸虫) in der Zeitspanne 1990-2006 lieferte 10'244 Treffer bei CNKI und 5'975 bei VIP. Wir beurteilten die 10 chinesischen biomedizinischen Fachzeitschriften welche die höchste Anzahl an Original-Arbeiten zum Thema Schistosomiasis

veröffentlichten hinsichtlich von Kriterien wie Sprachen und freiem Zugang. Die Mehrzahl dieser Fachzeitschriften wird auf Chinesisch veröffentlicht, doch sind oft englische Zusammenfassungen verfügbar. Freien Zugang zu den ganzen Artikeln gestatteten *China Tropical Medicine* in den Jahren 2005/2006, und seit 2003 das *Chinese Journal of Parasitology and Parasitic Diseases*; keine der anderen Fachblätter bot freien Zugang an. Wir geben einen Überblick über (i) die Entdeckung und Entwicklung von Medikamenten zur Behandlung von Schistosomiasis, und den Fortschritt innerhalb der letzten 20 Jahre im Bereich (ii) der chemischen Schneckenbekämpfung und (iii) des Umweltmanagements zur Kontrolle der Schistosomiasis in China. Unsere Schlussfolgerung ist, dass in der chinesischen Literatur wichtige Forschungsergebnisse von Bedeutung für lokale Kontrollmassnahmen und für den globalen Wissenskorpus veröffentlicht werden. Freier Zugang zu diesen Fachzeitschriften sollte gefördert werden und Sprachbarrieren entfernt, damit die wissenschaftliche Gemeinschaft die Fülle der chinesischen Forschung in Zukunft noch besser nutzen kann.

(Übersetzung: Peter Steinmann)
