# Supplementary material for: Harnessing the wealth of Chinese scientific literature: schistosomiasis research and control in China
Source: Emerg Themes Epidemiol. 2008 Sep 30;5:19. doi: 10.1186/1742-7622-5-19 (PMC2576166; doi:10.1186/1742-7622-5-19)
Supplement: Additional File 5 — Abstract in Spanish. [file 1742-7622-5-19-S5.pdf]

Spanish / Español

Perspectiva Analítica

Capturando la riqueza de la literatura científica China: investigación y control de la esquistosomiasis en China.

Autores: Qin Liu, Li-Guang Tian, Shu-Hua Xiao, Zhen Qi, Peter Steinmann, Tippi Mak, Jürg Utzinger, Xiao-Nong Zhou

Resumen

Así como la economía china sigue creciendo, también lo hacen su investigación en ciencias biomédicas y las actividades de publicación relacionadas con este medio. Las llamadas enfermedades tropicales desatendidas que ocurren mayoritariamente en países en vías de desarrollo son persistentes, e incluso emergentes, en ciertas zonas de China. Este artículo se propone documentar el potencial de investigación presente en las bases de datos bibliográficas en biomedicina en China. La contribución de China a la epidemiología y el control de la esquistosomiasis es una excelente ilustración. Para este artículo, buscamos en dos bases de datos comúnmente utilizadas: la *China National Knowledge Infrastructure* (CNKI) y la *VIP Information* (VIP). Usando la palabra clave “Schistosoma” (血吸虫) y abarcando el periodo de 1990 a 2006, obtuvimos 10,244 resultados en la INCC y 5,975 en la VIP. A continuación, identificamos las diez

revistas chinas de biomedicina que publicaron el número más alto de investigación original en esquistosomiasis y las examinamos bajo ciertos criterios, entre estos los idiomas de publicación y el libre acceso. Aunque la mayoría de las revistas son publicadas en chino, generalmente disponen de un resumen en inglés. Libre acceso a los artículos de la revista *China Tropical Medicine* fue ofrecido para el periodo 2005/2006 y para la revista *Chinese Journal of Parasitology and Parasitic Diseases* desde el 2003. Ninguna de las demás revistas estudiadas ofrece libre acceso. Investigamos (i) el descubrimiento y desarrollo de fármacos anti-parasitarios, (ii) el progreso en cuestiones de plaguicidas, y (iii) la gestión del medio ambiente para el control de la esquistosomiasis en China en los últimos 20 años. En conclusión, una cantidad considerable de investigación es publicada en la literatura científica china, la cual es relevante para medidas locales de control y para el conocimiento científico global. El libre acceso necesita ser promovido y los obstáculos del idioma eliminados para que la riqueza de la investigación china pueda ser apreciada por la comunidad científica.

(Traducido por Annick Bórquez)
