# Supplementary material for: Harnessing the wealth of Chinese scientific literature: schistosomiasis research and control in China
Source: Emerg Themes Epidemiol. 2008 Sep 30;5:19. doi: 10.1186/1742-7622-5-19 (PMC2576166; doi:10.1186/1742-7622-5-19)
Supplement: Additional File 6 — Additional Table 1. Characteristics of the 10 Chinese journals publishing the highest number of original schistosomiasis research articles. [file 1742-7622-5-19-S6.pdf]

Additional Table 1. Characteristics of the 10 Chinese journals publishing the highest number of original schistosomiasis research articles

Journal	Year of first publication	Language		Peer-review	Databases	Open access	Core journal	Type of articles published			
		Abstract	Main body	1=Original rese<br>2=Review<br>3=Short report	1=CNKI<br>2=VIP<br>3=WanFang<br>4=PubMed			Original re	Review	Short report	Letters
Chinese Journal of Schistosomiasis Control (中国血吸虫病防治杂志)	1989	Chinese and l	Chinese	1, 2, 3	1, 2, 3	No	Yes	70.8%	16.1%	11.3%	1.8%
Journal of Tropical Diseases and Parasitology (热带病与寄生虫学)	1994	Chinese and l	Chinese	1, 2, 3	1, 2, 3	No	Yes	77.2%	13.3%	7.1%	2.4%
Chinese Journal of Parasitology and Parasitic Diseases (中国寄生虫学与寄生虫病杂志)	1983	Chinese and l	Chinese or	1, 2, 3	1, 2, 3,4	Yes (since :	Yes	81.2%	6.2%	6.9%	5.7%
Parasitoses and Infectious Diseases (寄生虫病与感染性疾病 (实用寄生虫病杂志))	1993	Chinese and l	Chinese	1, 2, 3	1, 2, 3	No	Yes	84.2%	4.6%	2.9%	8.3%
Journal of Pathogen Biology (中国病原生物学杂志 (中国寄生虫病防治杂志))	1988/9	Chinese and l	Chinese or	1, 2, 3	1, 2, 3	No	Yes	75.5%	8.8%	10.8%	4.9%
Chinese Journal of Zoonoses (中国人兽共患病学报 (中国人兽共患病杂志))	1985	Chinese and l	Chinese	1, 2, 3	1, 2, 3	No	Yes	76.6%	15.1%	5.2%	3.1%
International Journal of Medical Parasitic Diseases (国际医学寄生虫病杂志 (国外医学寄生虫病分册))	1973	Chinese and l	Chinese or	1, 2, 3	1, 2, 3	No	No	30.2%	60.5%	3.8%	5.5%
Journal of Public Health and Preventive Medici (公共卫生与预防医学 (湖北预防医学杂志))	1990	Chinese and l	Chinese	1, 2, 3	1, 2, 3	No	No	90.8%	3.4%	1.3%	4.5%
Practical Preventive Medicine (实用预防医学)	1994	Chinese and l	Chinese	1, 2, 3	1, 2, 3	No	Yes	79.9%	2.4%	16.2%	1.5%
China Tropical Medicine (中国热带医学)	2001	Chinese and l	Chinese or	1, 2, 3	1, 2, 3	Yes (2005,	Yes	85.4%	5.9%	1.1%	7.6%
